# Irisin, an Effective Treatment for Cardiovascular Diseases?

**DOI:** 10.3390/jcdd9090305

**Published:** 2022-09-13

**Authors:** Chen Liu, Aili Wei, Tianhui Wang

**Affiliations:** 1 Tianjin Key Lab of Exercise Physiology & Sports Medicine, Tianjin University of Sport, Tianjin 301617, China; 2 Institute of Environmental and Operational Medicine, Academy of Military Medicine Sciences, Academy of Military Sciences, Tianjin 300050, China

**Keywords:** myokine, biomarker, irisin, cardiovascular diseases

## Abstract

Irisin, as one of the myokines induced by exercise, has attracted much attention due to its important physiological functions such as white fat browning, the improvement in metabolism, and the alleviation of inflammation. Despite the positive role that irisin has been proven to play in the prevention and treatment of cardiovascular diseases, whether it can become a biomarker and potential target for predicting and treating cardiovascular diseases remains controversial, given the unreliability of its detection methods, the uncertainty of its receptors, and the species differences between animals and humans. This paper was intended to review the role of irisin in the diagnosis and treatment of cardiovascular diseases, the potential molecular mechanism, and the urgent problems to be solved in hopes of advancing our understanding of irisin as well as providing data for the development of new and promising intervention strategies by discussing the causes of contradictory results.

## 1. Introduction

Cardiovascular diseases (CVDs) are the general term for cardiovascular and cerebrovascular diseases that refer to hemorrhagic or ischemic diseases of the brain, heart, and other tissues or organs caused by hypertension, atherosclerosis, and hyperlipidemia. In addition, the prevalence of cardiovascular diseases is reportedly rising in China [1], which is facing the dual pressure of population aging and the prevalence of metabolic risk factors. The increasing incidence of cardiovascular diseases has become the leading cause of death among residents. In 2019, CVDs accounted for 46.74% and 44.26% of deaths in rural and urban areas, respectively, thus intensifying the economic burden of CVDs on society [2]. Therefore, it is urgent to strengthen the prevention and control of cardiovascular diseases.

The main treatment regimens for cardiovascular diseases are drug therapy, interventional therapy, and surgery, with drug therapy as the most widely used. Drugs that are commonly used to treat cardiovascular diseases include calcium channel blockers (CCBS), angiotensin converting enzyme inhibitors (ACEI), angiotensin converting enzyme antagonists (ARBs), vasodilators, and statins [3]. Despite the excellent efficacy of these drugs, prolonged use has caused serious adverse reactions in patients [4]. These inadequacies suggest that the development of new methods and strategies for the prevention and treatment is of critical clinical importance.

Studies have shown that exercise can play a positive role in regulating the shape and function of the heart and arteries, which can be used as one of the important means of non-drug therapy for CVDs [5,6,7]. Based on the beneficial effects of exercise through specific pathways and cellular processes, scientists have proposed an emerging therapeutic approach called “exercise mimetics” [8], designed to enhance or mimic the beneficial effects of physical exercise for disease prevention and relief [8], which is why the mechanisms by which exercise prevents and treats cardiovascular diseases need to be elucidated. So far, a large number of studies have shown that exercise can contribute to the treatment of cardiovascular diseases by increasing the expression of irisin [9,10,11]. Research has indicated that irisin has the potential to be an important biomarker and potential target for the evaluation, prevention, treatment, and rehabilitation of some CVDs. However, due to the heterogeneity of the experimental design and methods, there are some differences and contradictions between the different research results. In light of these controversies and the potential functions of irisin, the elucidation of the role of irisin in CVDs may help to identify new and effective targets for the screening, diagnosis, and treatment of CVDs. This review aimed to outline the effects of irisin on the diagnosis and treatment of CVDs.

## 2. FNDC5 and Irisin

Irisin, as a myokine secreted by skeletal muscle, has attracted much attention since its discovery in 2012. Irisin is cleaved from its precursor protein fibronectin type III domain containing protein 5 (FNDC5). However, FNDC5 was inhibited by peroxisome proliferator-activated receptor γ coactivator-1α (PGC1-α) [12]. When exercising or being stimulated by cold, skeletal muscle can contract and activate PGC1-α to indirectly upregulate the expression of FNDC5 and stimulate the production and secretion of irisin [13]. Although numerous studies have shown that FNDC5 can be cleaved into irisin, the mechanism by which FNDC5 is regulated and cleaved to generate irisin remains unknown. As the precursor of irisin, FNDC5 is expressed not only in skeletal muscle, but in other tissues and organs of the body, especially in the heart and brain. Although FNDC5 is distributed in many organs of the body, skeletal muscle secretes 72% of the total irisin [14]. Irisin is highly conserved among species, with 100% sequence identity between mice and humans [12,15]. Notably, the promoter encoding the precursor FNDC5 protein in humans is different from that in other species, which is ATG, whereas in humans, it is mutated to ATA [16]. Studies have shown that the ATA initiation density is usually associated with low translation efficiency of eukaryotic mRNA [17], suggesting that there are different translation mechanisms and efficiency among humans. This difference casts doubt on the possibility that the various positive effects observed in animal models including the promotion and mechanism of irisin by exercise can also be found in humans.

PGC1-α upregulation can stimulate FNDC5 expression, and ATP deprivation in intracellular muscle after exercise may trigger FNDC5 synthesis and irisin release [18]. In contrast, the transforming growth factor-β (TGFβ) effector protein SMAD (drosophila mothers against decapentaplegic) Family Member 3 (SMAD3) reduces the expression of FNDC5 and PGC1-α by binding to their promoter regions, negatively affecting the production and secretion of irisin [19].

## 3. Physiological Function of Irisin

Since its discovery, irisin has appealed to researchers because of its important physiological function. Irisin, secreted by skeletal muscle, enters the circulatory system and reaches target organs, where it exerts its physiological effects by binding to αVβ5 integrin receptors in the bone and adipocytes [20]. The active structure of irisin appears to be a compact dimer [15] that is not affected by glycosylation [21]. Irisin has been shown to have ameliorative effects on a variety of diseases including cancer, obesity, diabetes, and cardiovascular diseases [22,23,24]. Studies have found that irisin has physiological effects in improving energy metabolic balance, enhancing cellular homeostasis by optimizing autophagy, promoting mitochondrial quality control, reducing reactive oxygen species (ROS) production, and alleviating inflammation [25,26,27]. The exploration of the role of irisin in energy metabolism such as the activity in adipocytes, muscle cells, and bone cells has further clarified the relationship between irisin and energy metabolism [28]. Studies have shown that irisin therapy alleviates ROS accumulation and autophagy by targeting uncoupling protein 2 (UCP2) [29]. Inflammation accelerates the progression of cardiovascular disease that often involves endothelial dysfunction. Irisin reduces inflammation through the p38 mitogen-activated protein kinase (P38 MAPK) pathway while improving endothelial dysfunction [30].

## 4. Regulation of Irisin by Exercise

A large number of studies have demonstrated that exercise can improve skeletal muscle irisin secretion, and its secretion is affected by the type, intensity, frequency, and duration of exercise [31,32,33]. A study based on acute exercise in humans showed that both endurance and resistance exercise increased the irisin levels in the blood, and resistance exercise is more likely to cause changes in irisin than endurance exercise [34]. For a single exercise, both low-intensity and high-intensity exercise can induce the secretion of irisin, and high-intensity exercise can induce more irisin production [34]. Some studies have shown that low-intensity aerobic exercise does not induce a significant increase in serum irisin [35]. However, for long-term exercise, no increase in the blood irisin level or changes in PGC-1α and FNDC5 were observed during 21 weeks of endurance exercise. In the study of 16-week aerobic exercise, irisin in the blood increased significantly after exercise [20,36,37]. Furthermore, in another study, the resting irisin levels did not differ between healthy women who trained for 0 and 6 weeks, but the blood irisin levels significantly increased by 9.5% and 18.15%, respectively, after a vigorous exercise session [38]. The results showed that acute but not chronic exercise stimulated an increase in irisin levels. However, given that the increased level of irisin after 6 weeks of training was much higher than that after 0 weeks of training, it suggests that long-term exercise may increase the sensitivity of the body to secrete irisin, but more studies are needed to prove this.

At present, the results of the exercise-induced increase in irisin are controversial, which may be related to the heterogeneity of the experimental design, detection method, sampling time, experimental population type, and exercise mode. The half-life in the body is only 1 h [18], Therefore, in future research, we should mainly use the time after exercise. The results vary, as shown in Table 1.

## 5. Irisin and CVDs

### 5.1. Irisin, Biomarker for CVD Diagnosis

Irisin can be used as a biomarker for the risk diagnosis of CVDs, to some extent. Studies have shown that circulating irisin concentrations are inversely associated with cardiovascular health risk factors such as hyperglycemia, triglycerides, visceral fat, and extracellular lipid deposition [47,48,49], but negatively correlated with risk factors of cardiovascular health such as hyperglycemia, triglycerides, visceral adiposity, and extramyocellular lipid deposition [47].

Circulating irisin levels were decreased in patients with CVDs. After adjusting for age and behavioral factors, serum irisin concentrations were inversely associated with the prevalence of coronary artery calcification (CAC) in Japanese males aged 40 to 79 [50]. Multivariate logistic regression analysis showed that serum irisin concentrations were negatively correlated with the atherosclerosis indices [51]. Similarly, some studies have shown that the severity of coronary heart disease corresponds to the serum irisin levels in patients with stable angina pectoris, suggesting that serum irisin can be used to predict the severity of coronary heart disease [52]. In addition, a meta-analysis based on 741 studies involving 876 coronary artery disease (CAD) patients and 700 controls reported that circulating irisin concentrations were reduced by 18.10 ng/mL in patients with coronary heart disease or atherosclerosis compared with the healthy controls [53]. In addition, serum irisin concentrations in type 2 diabetic women were significantly lower than in the normal controls, while the serum irisin concentrations in diabetic patients with atherosclerosis were lower than in diabetic patients without atherosclerosis [54]. Circulating irisin levels were lower in hypertensive patients and inversely correlated with systolic/diastolic blood pressure [55].

After treatment, the circulating irisin levels recovered. In 85 patients with primary hypertension, valsartan plus amlodipine was administered for 12 weeks, and the irisin levels were significantly elevated [56]. After 12 weeks of medication with valsartan and amlodipine, the serum irisin levels in hypertensive patients also increased significantly [56].

Irisin can also be used to predict prognosis. A study in which serum irisin concentrations were assessed by the enzyme-linked immunosorbent assay in 102 Parkinson′s disease (PD) patients and 35 age- and sex-matched controls demonstrated that serum irisin was significantly associated with sarcopenia and carotid atherosclerosis in peritoneal dialysis patients [57]. A higher level of irisin in patients with heart failure was associated with more deaths than a lower level of irisin [58]. Studies have shown that irisin levels are usually high in heart failure patients with cachexia [59], but low in female patients [60]. In a prospective single-center cohort study, the association between irisin concentrations and adverse cardiovascular events during a 3-year follow-up was assessed [10]. The research showed that serum concentrations of irisin were elevated in post-ST-elevation myocardial infarction patients who were at higher risk of adverse cardiovascular events. This study revealed that long-term high basal levels of irisin may have adverse effects. It can be concluded that irisin may have a double-edged sword effect on energy metabolism and tissue repair.

In conclusion, in some cases, irisin can be used as a biomarker for the risk of CVDs. However, due to the different methods of the detection of irisin, there are contradictory and controversial findings, so more research is needed to explore the mechanism.

### 5.2. The Regulation of Irisin on CVDs

#### 5.2.1. Hypertension

Hypertension is an important risk factor for cardiovascular diseases, while exercise is one of the best ways to combat hypertension [61]. Exercise can also improve the expression level of irisin in the treatment of hypertension, so it can be speculated that the expression level of irisin is related to the severity of hypertension. Irisin levels were higher in hypertensive patients than in healthy people and were positively correlated with systolic blood pressure [62]. A study was conducted on spontaneous hypertensive rats (SHRs) and Wistar–Kyoto rats with normal blood pressure, and irisin was injected intravenously to the rats. The results showed that the blood pressure of the rats decreased [63], indicating that irisin could lower hypertension.

Experiments have shown that endothelial cells (ECs) produced by irisin can increase the expression and phosphorylation of endothelial nitric oxide synthase (eNOS) but maintain normal blood pressure by activating the PI3K (phosphatidylinositol 3-kinases)/Akt(protein kinase B) pathway [64], and that irisin can reduce blood pressure in spontaneously hypertensive rat models by activating the nuclear factor red like 2 related factor 2 (Nrf2) pathway and sympathetic regulation of paraventricular nucleus of the hypothalamus [63]. Other studies have confirmed that FNDC5 overexpression reduces blood pressure, and FNDC5 plays a beneficial role in vascular remodeling and improving blood pressure [65]. Intravenous irisin can activate Nrf2, correct neurotransmitter imbalance in the paraventricular nucleus (PVN), and exert an antioxidant function to effectively inhibit spontaneous hypertension in rats [63]. It has been found that changes in blood pressure are also associated with injection sites. Central irisin injection can promote systolic blood pressure, while peripheral irisin injection can reduce blood pressure [66].

It has also been found that AMP (adenosine monophosphate)-activated protein kinase (AMPK) activated by irisin can phosphorylate Akt and eNOS with increased nitric oxide (NO) production [67]. Irisin improves endothelial function through the AMPK/eNOS pathway [68] and results in lowered blood pressure. Hypertension can cause myocardial hypertrophy. When the AMPK-ULK1 (Unc-51 Like Autophagy Activating Kinase 1) signaling pathway is activated by irisin, protective autophagy and autophagy flux can be induced to prevent cardiac hypertrophy caused by pressure overload [69] and reduce the risk of high blood pressure. Irisin injection has the antihypertensive effect in hypertensive rats by reducing oxidative stress and inflammatory response.

Irisin produced by skeletal muscle can lower blood pressure [70], however, the mechanism is not fully understood. What is worse, there are some contradictory reports. Recent studies have done nothing more than reveal the therapeutic and protective effects of irisin against hypertension, but a clear conclusion that irisin can help prevent hypertension is lacking. Despite the simple symptoms of hypertension, its pathogenesis is far from conclusive. The benefits of exercise can reduce drug intake without reducing drug resistance, which can lower blood pressure and keep patients healthy.

Therefore, an intimate knowledge of the molecular mechanism of irisin in hypertension is the key to lowering the incidence of cardiovascular diseases.

#### 5.2.2. Atherosclerosis

Atherosclerosis is a chronic inflammatory disease that is the most common pathogenesis of coronary heart disease (CHD) and many vascular diseases [71]. Coronary artery spasm or atherosclerosis can lead to pathological changes in the function or structure of the heart. Lesions are characterized by lipid aggregation, fibrous hyperplasia, and calcium deposition, followed by gradual degeneration and calcification of the middle layer of the artery, and finally by internal bleeding, plaque rupture, and local thrombosis [71]. Pearson correlation analysis showed that in the case of atherosclerosis, the serum irisin levels were negatively correlated with the severity of atherosclerosis [72], and the irisin levels showed varying degrees of fluctuation with the aggravation of the disease. Irisin proved to protect the endothelial cells and reduce or delay the occurrence and development of atherosclerosis [73]. Previous studies have found that irisin reduces atherosclerosis in apolipoprotein E-deficient mice by inhibiting ox-LDL (oxidized low-density lipoprotein)-induced cellular inflammation and apoptosis [74]. Furthermore, the supplementation of exogenous irisin has been shown to ameliorate AGE (advanced glycation end product)-induced inflammation and endothelial dysfunction by inhibiting ROS-NLRP3 (NOD-like receptor family pyrin domain containing 3) inflammasome signaling, and changes the progression of atherosclerosis [75]. This study can start people thinking along a new line about the direct treatment of atherosclerotic diseases.

Studies have found that exogenous irisin (0.01, 0.1, 1 μg/mL), as an intervention agent, significantly reversed AGE-induced oxidative stress and pyrin domain-containing-3 inflammasome signaling activation in a dose-dependent manner, and increased the production of eNOS and NO. The progression of atherosclerosis was improved [75].

According to some studies, irisin can reduce atherosclerotic plaque, lipid deposition, and macrophage aggregation but upregulate miRNA-126-5P dependent on extracellular signal-regulated kinase (ERK) [76]. Zhu et al. proved that irisin can reduce endothelial dysfunction by inhibiting oxidative stress and inhibit protein kinase C-β (PKC-β)/nicotinamide adenine dinucleotide phosphate (NADPH) oxidase and nuclear factor-kappa B (NFκB)-induced nitric oxide synthase [77], thereby reducing oxidative stress and the apoptosis of endothelial cells and delaying the onset of atherosclerosis. Studies have confirmed that irisin can reduce the recruitment of T lymphocytes and macrophages in atherosclerotic lesions [73]. After irisin treatment, the expressions of inflammatory mediators such as mRNA and protein intercellular adhesion molecule-1 (ICAM-1), vascular cellular adhesion molecule-1 (VCAM-1), monocyte chemoattractant protein-1 (McP-1), IL-6, and NF-κB were significantly decreased [78]. The mechanism of this process may be the mediated inhibition of the ROS-P38MAPK-NFκB signaling pathway and activation of the AMPK-PI3K-PKB (protein kinase B)-eNOS signaling pathway [73,74]. Irisin can significantly reduce the lesion size of the aorta through the ROS-P38 MAPK-NFκB signaling pathway and improve endothelial function [73]. In addition, irisin is used to treat atherosclerosis through both the endothelium-independent pathway and endothelium-dependent pathway to keep blood vessels healthy. Intraperitoneal injection of follistatin (FST) can promote the secretion of irisin in subcutaneous fat through the AMPK-PGC1-α-irisin signaling pathway, induce the browning of WAT (white adipocytes tissue), and activate the insulin pathway in beige fat, thus promoting metabolism [79]. Therefore, there is reason to speculate that FST can improve the process of some metabolic diseases such as atherosclerosis, while promoting the secretion of irisin. The specific mechanism needs to be further verified by experiments.

In the process of treating atherosclerosis with irisin, clinicians are puzzled about whether irisin directly leads to the expansion and contraction of blood vessels. There is little known on the mechanism involved in the regulation of blood vessels. Studies on the vasodilatory effects of irisin are too few to draw any definite conclusions. Therefore, a more elaborate design is needed in future studies to verify the vasodilatory effect of irisin in experimental animal models in order to explain the mechanisms involved in these signaling pathways. Irisin is an effective means by which to treat atherosclerosis. There are many reports that FNDC5 generates irisin after being cleaved.

#### 5.2.3. Heart Failure

As a main epidemic related to cardiovascular diseases in the 21st century, cases of heart failure are estimated to exceed 25 million worldwide and keep rising [80]. Heart failure is the terminal stage of many cardiovascular diseases with high mortality and is associated with impaired energy production, conduction, or utilization of the heart. Patients with advanced heart failure often present with reduced exercise tolerance, skeletal muscular dystrophy, and even cardiac cachexia. FNDC5, a precursor of irisin, has been shown to be downregulated in the skeletal muscle cells in patients with heart failure [81]. Studies have shown that in rat models of chronic heart failure, the irisin levels decrease with the development of heart failure [82].

Wang et al. administered exogenous irisin to a rat model of myocardial infarction and found that irisin protected the heart from ischemia/reperfusion (I/R) damage in a dose-dependent manner [83]. Liao et al. administered irisin for two weeks in a mouse model of myocardial infarction and found that irisin improved cardiac function, attenuated ventricular dilation, and reduced the area of infarction four weeks after myocardial infarction [84].

Irisin has been proven to be effective for heart failure. Studies have shown that αV integrin receptor promotes the physiological effects of irisin [20], and that irisin can increase the survival rate of heart failure patients by upregulating ROS, lactate dehydrogenase (LDH), and histone deacetylase 4 (HDAC4). The P38MAPK pathway and NFκB can reduce the inflammatory response of patients with heart failure. The regulation of caspase-9, caspase-3, mitochondrial permeability transition pore (mPTP), and ROS can significantly reduce the inflammatory response in patients with heart failure. One study proved that during the development of heart failure, irisin induced a protective autophagy and autophagy flow through the activation of the AMPK-ULK1 signaling pathway [69] to prevent both myocardial hypertrophy induced by excessive pressure and heart failure. Li et al. studied the effects of irisin on myocardial hypertrophy in different models including angiotensin II (Ang II) or phenylephrine (PE) treated cardiomyocyte or mouse aortic transverse contraction (TAC) models and demonstrated that irisin regulated autophagy via mTOR (mechanistic target of rapamycin) independent activation of the AMPK-ULK1 pathway, thereby relieving myocardial hypertrophy [69,85]. In addition, FNDC5 expression in the skeletal muscle is also correlated with the aerobic performance of heart failure patients [81]. The data suggest that FNDC5 may be considered as a promising biomarker and therapeutic target for heart failure [86].

**Figure 1:**
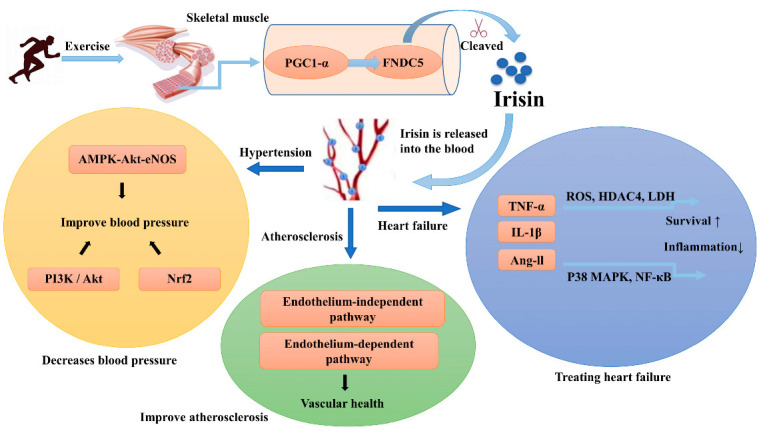
The mechanisms of irisin release on cardiovascular disease after exercise. Irisin, mainly secreted by muscle, may have effects on multiple organs and tissues; ROS—reactive oxygen species; P38 MAPK—p38 mitogen-activated protein kinase; PGC1-α—peroxisome proliferator-activated receptor-gamma coactivator-1α; FNDC5—fibronectin type III domain containing protein 5; Ang-ll—Angiotensin Ⅱ; TNF-α—Tumor necrosis factor-α; IL-1β—Interleukin-1; NFκB—nuclear factor-kappa B; LDH—lactate dehydrogenase; HDAC4—histone deacetylase 4; Nrf2—Nuclear factor erythroid 2-related factor 2; PI3K—phosphatidylinositol 3-kinases; Akt—protein kinase B; eNOS—endothelial nitroxide synthase.

While it is clear that irisin can protect the heart and improve cardiac function, research on the signaling pathways and mechanisms of action is still incomplete. In future studies, it is hoped that irisin can be used as a biological detection indicator for patients with heart failure, thus facilitating the diagnosis of severity and treatment of heart failure. The mechanism of irisin on cardiovascular diseases is shown in Figure 1.

#### 5.2.4. Other Cardiovascular Diseases

In addition to the three typical cardiovascular diseases above-mentioned, arrhythmias, myocardial infarction, cardiac fibrosis, and stroke are also very dangerous cardiovascular diseases [30]. However, studies on irisin and arrhythmias are limited.

One recent study offered the possibility of curing tachyarrhythmia via the microinjection of irisin into the suspected core of visceral vagal neurons, inducing an increase in cellular Ca^2+^ concentration and neuronal depolarization, resulting in bradycardia in awake rats [87]. Deng et al. [88] found that overexpression of irisin and over-expressed FNDC5 on bone marrow mesenchymal stem cells (BM-Mscs) showed that irisin or FNDC5 could significantly reduce myocardial fibrosis. Previous studies have suggested that irisin could be a promising therapy against myocardial fibrosis as irisin attenuates angiotensin II-induced cardiac fibrosis via the Nrf2 mediated inhibition of the ROS/TGFβ1/Smad3/Smad2 (Smad2/3) signaling axis [89]. Some studies have suggested that irisin can upregulate the level of brain-derived neurotrophic factor (BDNF), which can protect nerve cells from damage during stroke [90].

As a new myokine, irisin has played an important role in the field of cardiovascular diseases in recent years. Due to its unique anti-inflammatory and anti-apoptotic properties, irisin has been widely used in the diagnosis of cardiovascular diseases. However, little research has been conducted on the mechanism of irisin action in cardiovascular diseases.

## 6. New Treatments and Strategies Based on Irisin

Irisin has proven to be of great value in the field of life sciences, and its potential therapeutic effect in metabolic diseases or metabolic health problems will lead to new uses in the treatment of obesity, type II diabetes, or cardiovascular diseases.

There are currently a wide range of drugs used in the clinical treatment of cardiovascular diseases, which can contribute to clinical research on the mechanism of action of related drugs and provide a useful reference [91]. As a target for the treatment of cardiovascular diseases, irisin may become a new drug for this purpose, while opening up new areas for research on drugs against cardiovascular diseases [28,92]. For example, irisin could be used as a functional food, drug, or a new target for the treatment of metabolic diseases or metabolism-related health problems. At the same time, strategies for the regulation of irisin upstream signaling pathways may also provide innovative ideas for the control over or intervention in human metabolic diseases or metabolic health problems.

Thus far, many studies have proven that drugs can induce the expression of FNDC5 and irisin. For example, LBP (low back pain) can induce the expression of FNDC5. Leptin can increase the expression of FNDC5 through negative feedback regulation. Icariin upregulates the expression of FNDC5 and irisin by activating the AMPK pathway. Recombinant human growth hormone (rhGH) significantly upregulates the level of irisin. Orlistat can upregulate the serum irisin levels, as shown in Table 2.

In conclusion, understanding the mechanism of irisin’s therapeutic effect is critical. However, the mechanism and pathway of irisin are far from clear. Therefore, future research should focus on elucidating the mechanism of irisin’s therapeutic effect, discovering new therapeutic targets based on the upstream and downstream of irisin, and developing drugs for clinical practice.

## 7. Conclusions and Prospects

There are currently many studies on the role of irisin in cardiovascular diseases. The results suggest that irisin may be a biological indicator and therapeutic target for the diagnosis of cardiovascular diseases. Both in vitro and in vivo experiments have proven its therapeutic effect against cardiovascular diseases. However, it is still too early for use as a biomarker and therapeutic target for detection, and many problems remain to be solved. There is no uniform standard for the quantitative detection methods of endogenous irisin; the commonly used methods are Western blot, ELISA, and mass spectrometry. Existing studies have shown that irisin concentrations obtained by different detection methods vary greatly, and studies have shown poor repeatability. The individual differences, half-life, sampling time, and sampling organization of irisin may affect the detection concentration of irisin, which may account for the inconsistency in the results. The detection of irisin in vivo remains a major challenge to researchers. Therefore, it is necessary to establish the detection standard of irisin in terms of the sampling time and detection methods in order to increase the reliability of the research results. In addition, although irisin is highly homologous among species, people have different initial passwords compared to other species. Therefore, researchers need to consider whether the conclusions based on animal models are suitable for human beings, and whether special transgenic animal models are needed to eliminate ethnic differences. Thus far, there have been many studies on the ability of irisin to improve cardiovascular disease, and some mechanisms have been elucidated, but there are still some key issues that remain unresolved. For example, the receptor of irisin is still unclear, as is the mechanism of how FNDC5 is cleaved into irisin. The elucidation of the related mechanisms is to precede the development of new therapeutic methods and strategies. Therefore, these problems are also the priorities of future research. It is also worth noting that studies have proven the protective effect of exogenous irisin against cardiovascular diseases in cells and animals, indicating the potential of irisin as a therapeutic target. However, due to the high blood concentration of irisin in previous studies, the dose of irisin that exerts biological effects is much higher than its physiological concentration. This raises new problems for irisin as a therapeutic target.

To sum up, there is still a long way to go before irisin can be used as a drug for the treatment of cardiovascular diseases and other diseases.

## Figures and Tables

**Table 1:** The effects of different exercise modes on the irisin levels in humans.

Intervention Mode	Exercise Frequency	Exercise Duration	Tissue	Detection Method	Change	Reference
Aerobic exercise	45 min/time	8 weeks	Blood	ELISA	irisin↑; Compared with pre-exercise	[39]
Aerobic exercise	40 min/time	16 weeks	Blood	ELISA	irisin↑; Compared with pre-exercise	[40]
Resistance exercise	60 min/time	12 weeks	Blood	ELISA	irisin↑; Compared with pre-exercise	[41]
Resistance exercise	55 min/time	13 weeks	Blood	ELISA	irisin↑; Compared with pre-training	[42]
Low-impact AE	1 h	/	Muscle	ELISA	irisin (no significance); Compared with pre- exercise	[35]
HIIT and moderate intensity continuous training	33, 41 min/time 3 times/week,	12 weeks	Blood	ELISA	irisin (no significance); Compared with pre- exercise	[43]
Resistance exercise (Progressive training)	/	12 weeks	Blood	ELISA	irisin (no significance); Compared with pre- exercise	[44]
A single strenuous endurance exercise	60 min/d	/	Blood	ELISA	irisin↑; Compared with pre- exercise	[45]
Resistance exercise	>60 min/d, 5 >times/week	8 weeks	Blood	ELISA	irisin↑; Compared with pre- exercise	[46]

ELISA—enzyme linked immunosorbent assay; AE—aerobic exercise; HIIT—high intensity interval training; ↑—promote.

**Table 2:** The effects of drugs on the irisin levels.

Model	Intervention	Detection Method	Change	Reference
Mouse	Leptin	PCR, ELISA	irisin↓FNDC5↑FNDC5 mRNA↑; compared to no injection	[93]
Mouse	Insulin	PCR, ELISA	irisin↓FNDC5 mRNA↓(muscle); compared to no injection	[93]
Mouse	Metformin	PCR	FNDC5 mRNA↓(muscle); compared to no injection	[93]
Mouse (Obesity)	Lcariin	PCR, WB, ELISA	PGC1-α↑ FNDC5mRNA↑ irisin↑; compared to no injection	[94]
Rat (DM)	BPS	ELISA	irisin↓; compared to no injection	[95]
Human (GHD)	rhGH	ELISA	irisin↑↑; compared to no injection	[96]
Human (SHT)	Levothyroxine sodium	ELISA	irisin↓; compared with those before treatment	[97]
Human (obesity)	Orlistat	ELISA	irisin↑; compared with those before treatment	[98]

FNDC5—fibronectin type III domain containing protein 5; PGC1-α—peroxisome proliferator-activated receptor-gamma coactivator-1α; DM—diabetes mellitus; BPS—Balanophora polysaccharide; rhGH—recombinant human growth hormone; GHD—growth hormone deficiency; SHT—subclinical hypothyroidism; ELISA—enzyme linked immunosorbent assay; PCR—polymerase chain reaction; ↑↑ indicates a significant improvement; ↑—promote; ↓—control.
